# A novel homozygous variant of *TMEM260* induced cardiac malformation and neurodevelopmental abnormality: case report and literature review

**DOI:** 10.3389/fmed.2023.1157042

**Published:** 2023-05-09

**Authors:** Mou Peng, Siyuan Jing, Sichen Duan, Guoyan Lu, Kaiyu Zhou, Yimin Hua, Tao Wang, Peng Yue, Yifei Li

**Affiliations:** ^1^Key Laboratory of Birth Defects and Related Diseases of Women and Children of MOE, Department of Pediatrics, West China Second University Hospital, Sichuan University, Chengdu, Sichuan, China; ^2^Department of Nursing, West China Second University Hospital, Sichuan University, Chengdu, China

**Keywords:** *TMEM260*, cardiac malformation, neurological disorder, case report, literature review

## Abstract

**Background:**

Congenital heart disease (CHD) represents the most widespread congenital birth defect among neonates worldwide, leading to substantial expenses and contributing significantly to premature death caused by birth defects. Despite the significance of CHD, research on its etiology remains limited and has failed to provide substantial evidence for the molecular basis of the disease. With the advancement of next-generation sequencing (NGS), genetic screening has become increasingly accessible, offering a greater capability for identifying potential genetic variants associated with CHD.

**Case presentation:**

Exome sequencing and variant analysis of *TMEM260* were performed to obtain genetic data, and clinical characteristics were determined. A complex and severe form of CHD, comprising a persistent truncus arteriosus type I, ventricular septal defect, right aortic arch, as well as critical neurodevelopmental delay and neurological dysfunction, was observed in a patient. This proband presented global muscle hypotonia and a significant delay in gross and fine motor development. Cranial computed tomography scanning showed the presence of bilateral apical, occipital, and temporal subdural effusions; slightly wider bilateral lateral ventricles and annular cisterns; and bilateral cerebral hemispheric parenchyma atrophy. Upon genetic analysis of the patient, a novel homozygous mutation was identified in the *TMEM260* gene. The mutation, c.1336_1339DEL, was found to be homozygous and resulted in a frameshift mutation, causing a p.L447Vfs^*^9 amino acid change. This mutation led to the deletion of a TCTC sequence from positions 1336 to 1339 in the *TMEM260* gene, changing leucine to valine at amino acid 447 and introducing a stop codon after the ninth amino acid. This structural deletion in the *TMEM260* protein resulted in the loss of gene function.

**Conclusion:**

This case report presents a newly discovered variant site in the *TMEM260* gene and reinforces the relationship between *TMEM260* molecular function and differentiation of mesoderm and ectoderm. Furthermore, our findings broaden the spectrum of variants in the *TMEM260* gene and contribute to advancing the genetic understanding of CHD.

## 1. Introduction

Congenital heart disease (CHD) is the most widespread congenital birth defect among living neonates globally, incurring substantial expenses (1). CHD also accounts for the majority of premature deaths due to birth defects (2). Advances in surgical techniques, hybrid interventional strategies, fetal cardiac interventions, and intensive care have significantly improved the survival rate of severe CHD cases (2). Despite these advancements, the etiology of CHD remains poorly understood and the molecular basis of CHD is yet to be fully established. The origins of CHD are thought to result from a combination of adverse environmental exposures and specific genetic abnormalities, such as exposure to harmful molecules such as di-(2-ethylhexyl)-phthalate and alcohol. The American Heart Association published a scientific statement in 2018 on the genetic basis of CHD, summarizing the latest information on genetic and locus variants associated with CHD, but our understanding of the molecular basis of CHD remains limited (3–5).

Next-generation sequencing (NGS) technology has made it easier to obtain genetic screening results and identify potential genetic variants of CHD. One such gene is *TMEM260* (transmembrane protein 260), a protein-coding gene located on chromosome 14. Despite its presence, the function of *TMEM260* remains unclear, with no available research providing evidence of its role in global development through conventional or conditional genetic knock-out models. Currently, only two case reports have shown an association between mutations in *TMEM260* and severe CHD, combined with renal or neurological malformations, differentiated from mesoderm and ectoderm. The first report described four individuals from two families with biallelic truncating *TMEM260* variants presenting brain, cardiac, and renal abnormalities (6). The second report described eight individuals from five families with biallelic *TMEM260* variants suffering from severe structural heart defects and renal anomalies syndrome (7).

This study presents a case with a novel homozygous pathogenic variant of the *TMEM260* gene (c.1336_1339delTCTC; p.L447Vfs^*^9), displaying significant cardiac developmental abnormality and cerebral atrophy. This case report provides new information on a variant site in the *TMEM260* gene and strengthens the association between the molecular function of *TMEM260* and mesoderm and ectoderm differentiation. The clinical phenotypes of *TMEM260* variants are restricted to truncus arteriosus, providing a useful model to uncover the mechanisms involved in truncus arteriosus.

## 2. Case presentation

### 2.1. Clinical presentation and physical examination

This research has been approved by the Ethics Committee of the West China Second Hospital of Sichuan University (approval no.2014-034). Informed consent was obtained from the patient's parents prior to performing whole-exome sequencing (WES) and for the inclusion of the patient's clinical and imaging details in subsequent publications.

This 8-month-old female infant was admitted to the hospital with a severe lung infection, presenting with a recurrent cough and shortness of breath. Additionally, the infant demonstrated a significant difficulty in feeding and had a body length and weight below the 3rd percentile for her age, indicating developmental retardation. Her parents reported poor performance in motor function and a significant neurodevelopmental delay, with acute critical illness, malnutrition, and thin subcutaneous fat. The infant's head circumference measured 39 cm and she had a square skull with a large anterior fontanel measuring 3 × 3 cm. Her visual and hearing evaluations were normal. The infant also exhibited high levels of muscle tension and a rupture and scab on the top of the head. Two lower incisors had erupted, and the pharynx was red with hoarseness in her voice. Respiratory movements were symmetrical in both lungs with rough breath sounds, significant wet rales heard bilaterally, and occasional wheezing sounds. The apex beat was located in the lower left, and there was a sense of lift in the precordial area, with an enlarged heart boundary and increasing heart rate, though the heart sound was dull, and a level II-III systolic murmur was recorded. The abdomen was soft with a medium-textured liver located 6 cm below the subcostal margin and 4–5 cm below the xiphoid process, and a non-palpable spleen. The first and second toes on the left foot were dry and black, with clear boundaries between them and surrounding healthy tissue. The muscle strength was reduced to grade 3 in the upper limbs and grade 2 in the lower limbs, indicating global muscle weakness. She was unable to sit independently and failed to handle grasp building blocks by 12 months of age. Moreover, she failed to produce pronunciation clearly. A Gesell evaluation conducted at 12 months revealed a significant delay in gross and fine motor development. No pathological or meningeal irritation signs were present. The parents reported no positive family history of cardiac attack or cardiovascular disease and declined genetic sequencing analysis and third-generation *in vitro* fertilization in the next pregnancy. The mother's first pregnancy was terminated at 28 weeks due to awareness of fetal tetralogy of Fallot.

### 2.2. Laboratory and imaging results

**Figure 1:**
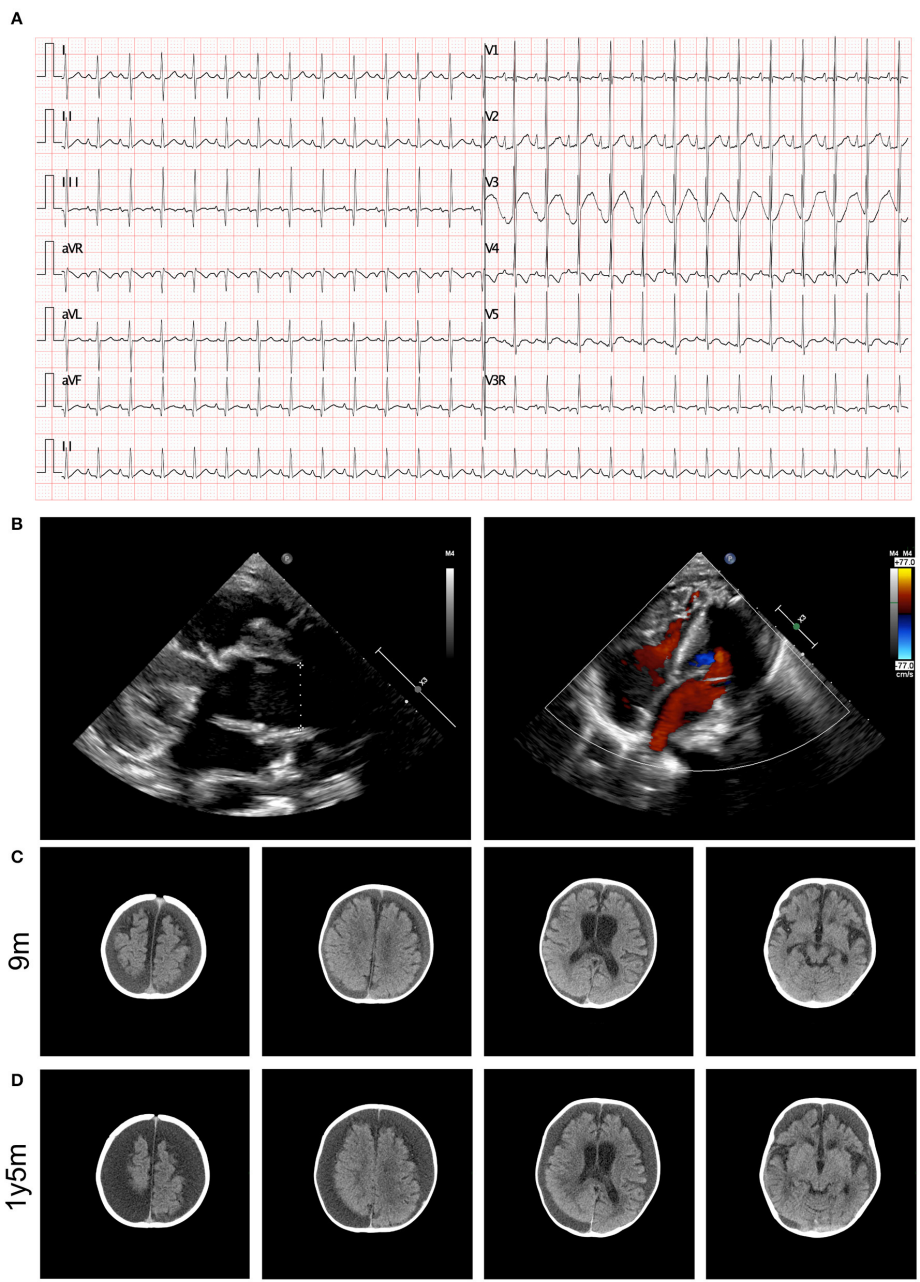
Clinical image presentation of the proband. **(A)** ECG recording demonstrated abnormal signals in right atrial, suspected right ventricular hypertrophy, sinus tachycardia, and ST segment changes (lead I, II, III, aVF, and V3–V6 depression of 0.1–0.5 mV and aVR and V1 increase by 0.1–0.2 mV). **(B)** Echocardiography revealed type I truncus arteriosus. **(C)** Cerebral CT examination demonstrated bilateral cerebral hemispheric parenchyma atrophy at 9 months old. **(D)** Cerebral MRI identified aggressive atrophy at 12 months old.

The results of a blood gas analysis indicated the presence of metabolic acidosis, with a pH of 7.278, a BE of −11.8 mmol/L, and an ${\text{HCO}}_{3}^{-}$ level of 13.3 mmol/L. The results of blood biochemical tests showed elevated levels of B-type natriuretic peptide (1526.27 pg/mL, normal range <146 pg/mL). No significant findings were identified in the results of hepatic and renal function tests. Elevated levels of blood ammonia (108 umol/L) were noted. An electrocardiogram (ECG) revealed abnormal signals in the right atrium and suspected right ventricular hypertrophy, as well as sinus tachycardia and ST segment changes (depression in lead I, II, III, aVF, and V3-V6 of 0.1–0.5 mV and an increase in aVR and V1 of 0.1–0.2 mV), as illustrated in Figure 1A. Heart failure and cardiac malformation were suspected, leading to the immediate performance of an echocardiogram. The echocardiogram identified a severe type of CHD, type I truncus arteriosus, with ventricular septal defect (VSD) and patent foramen ovale (PFO) (Figure 1B). The aorta was found to be in the right position, with mild semilunar valve regurgitation. The neurological system was assessed through cranial computed tomography (CT) scanning, which showed the presence of bilateral apical, occipital, and temporal subdural effusions; slightly wider bilateral lateral ventricles and annular cisterns; and bilateral cerebral hemispheric parenchyma atrophy (Figure 1C). Chest CT scanning also identified the presence of pulmonary infection and enlargement of the left atrium and left ventricle, while CT scanning did not show any abnormalities in the kidneys.

### 2.3. Molecular results

**Figure 2:**
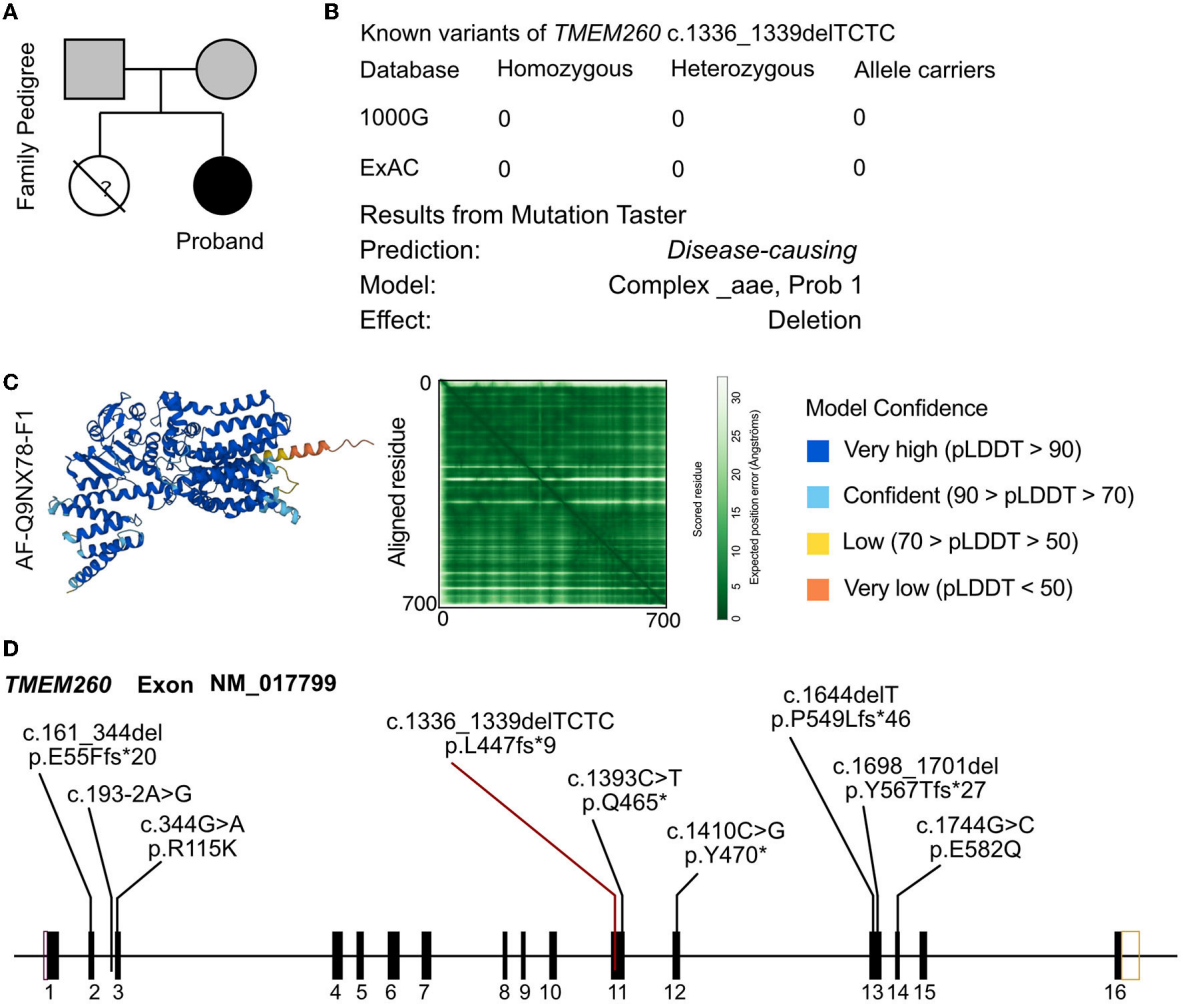
Molecular analysis of *TMEM260* variants. **(A)** Family pedigree of the proband. **(B)** The prevalence of the *TMEM260* variant identified in the proband. **(C)** Molecular structure based on the AF-Q9NX78-F1 template. **(D)** Reported variants of the *TMEM260* gene.

In the analysis of the proband, a novel homozygous mutation in the *TMEM260* gene was identified. The c.1336_1339DEL homozygous variant was found to result in a frameshift mutation in the amino acid sequence (p.L447Vfs^*^9). The parents of the proband were found to be carriers of the variant by WES analysis and did not exhibit any clinical manifestations (Figure 2A). No other cardiovascular-related variants were identified in the proband or her parents. The frequency of this mutation in the general population is not recorded in any database (Figure 2B). The identified frameshift mutation resulted in the deletion of a few bases (TCTC) from positions 1336 to 1339 in the *TMEM260* gene. This led to a change in amino acid 447 from leucine to valine and the creation of a stop codon after the ninth amino acid, causing a structural deletion in the *TMEM260* protein and resulting in the loss of gene function (Figure 2C). According to MutationTaster analysis, the variant was determined to be a disease-causing mutation with a probability value of 1.00. The frameshift deletion caused a complete disruption in the structure of the *TMEM260* protein, particularly in the transmembranous part, which impaired its normal molecular function. As the structure and function of the *TMEM260* protein are not well understood, the discovery of this pathogenic site is of great significance for advancing the understanding of this protein. A summary of all reported variants of *TMEM260* is presented in Figure 2D and Table 1.

**Table 1:** Genetic and clinical information for reported cases with variants of *TMEM260*.

	**Reported Proband**	**Ta-Shma et al. (6)**	**McQuillen et al. (7)**
Ethnicity	Chinese	Ashkenazi Jewish, Arabic	White British, Pakistani, Ashkenazi Jewish, Sudanese, Chinese
Reported variants	Homo: c.1336_1339delTCTC; p.L447fs^*^9	Homo: c.1393C>T; p.Q465^*^ c.1698_1701delCTAT; p.Y567Tfs^*^27	Homo: c.1410C>G; p.Y470^*^; two cases c.1698_1701delCTAT; p.Y567Tfs^*^27 Compound Heter: c.(344G>A):(161_ 344del); p.(R115K):(E55Ffs^*^20) c.(1393C>T):(1644delT); p.(Q465^*^):(P549Lfs^*^46); two cases c.(193-2A>G):(c.1744G>C); p.(?):(E582Q); two cases
Gender	Female	2 Males, 2 Females	5 Males, 3 Females
Deceased	NO	3/4 are deceased (6 weeks, 2 months, 1 year)	6/8 are deceased (3 of them had been terminated during pregnancy at 21 weeks, 22 weeks, 24 weeks; 3 of them dead postnatally at 3 months, 4 months, 5 years)
Cardiac defects			
Septal defects(s)	VSD	VSD 4/4, ASD 1/4	VSD 8/8, ASD 1/8
Truncus arteriosus	Type I	2/4	8/8
**TOF**	–	1/4	1/8
Pulmonary stenosis	–	–	2/8
Interrupted aortic arch	–	1/4	–
Right aortic arch	+	1/4	1/8
Tricuspid valve atresia	–	1/4	1/8
Partial anomalous	–	1/4	1/8
Meniscus regurgitation	Mild	N/A	N/A
Neurodevelopmental defects			
Agenesis of corpus callosum	–	–	2/3
Microcephaly	–	–	1/4
Developmental delay	Severe	1/8	N/A
Cerebral atrophy	Significant	N/A	N/A
Renal defects			
Renal failure	–	3/8	N/A
Anuria/Oliguria	–	1/8	2/4
Renal cysts	–	–	1/4
Horseshoe kidney	–	1/8	N/A
Limb defects			
Polydactyly	–	–	1/4
Overriding toes	–	–	1/4
Other			
Other features	Cardiac arrest	Hearing loss, upper respiratory tract infection requiring PICU, protein losing enteropathy, cyanosis, severe and prolonged chylothorax following surgery, vertical midline linear, skin defect over chest wall, single umbilical artery, left pre-auricular sinus necrotizing enterocolitis with perforation of transverse colon at day 10	

### 2.4. Final diagnosis, treatment, and follow-up

The infant was diagnosed with a severe and complex CHD of type I persistent truncus arteriosus, VSD, and a right aortic arch. The infant also displayed critical neurodevelopmental delay and neurological dysfunction. Despite undergoing a multi-phase surgical correction and receiving treatment with anti-infection measures, myocardial protection, inotropic agents, and mechanical ventilation, the patient suffered several cardiac arrests during hospitalization in the cardiac care unit. These were managed with prompt cardiopulmonary resuscitation.

Post-surgery, the patient developed severe antibiotic-resistant infections, including carbapenem-resistant *Acinetobacter baumannii* and *Elizabethkingia meningitidis*, and was treated with tigacycline, polymyxin, and sulperazon.

During follow-up, the patient presented with recurrent heart failure and pulmonary infections. Echocardiography showed dilation of the aorta, a small shunt through the interventricular septum, slightly accelerated forward blood flow through the artificial pulmonary valve with slightly strong ultrasound signals, moderate tricuspid regurgitation, and a slightly reduced left ventricular contractile function. The patient also experienced regression in motor performance, and a follow-up cranial CT scan revealed aggressive progression of cerebral atrophy (Figure 1D).

## 3. Discussion

The simultaneous development of the neurological and cardiovascular systems in the human fetus is a result of complex genetic programs, followed by periods of morphological refinement in response to physiological function (8). Given the interdependence of these developmental programs, it is believed that significant malformations of the cardiovascular system can impact the development of other organs (8). There is evidence that 28–59% of newborns with congenital heart defects experience some degree of brain injury prior to surgical correction, particularly in cases of severe CHD (9). Advances in functional magnetic resonance imaging analysis have also shown significant impairments in cerebral function in these patients (10). Recent studies have shed light on the shared genetic background of the neurological and cardiovascular systems. A GWAS study conducted by Zaidi et al. confirmed that the genetic basis of CHD can impact the development of other systems (11). Exome sequencing of 1,213 parent–offspring trios with CHD revealed a series of protein-damaging *de novo* mutations, particularly in genes that are highly expressed and shared in the developing heart and brain. These mutations were identified in nearly 20% of patients with CHD and extra-cardiac congenital anomalies, whereas only 2% of CHD patients with genetic variants showed no abnormalities in other systems. Truncus arteriosus is a rare type of CHD, with only a few genes identified to be associated with its formation. Currently, the genes *NKX2.5, NKX2.6, TBX1*, and *GATA6* have been retrieved from patients with truncus arteriosus. Previous studies have also indicated that variants of *TMEM260* may contribute to the phenotype of truncus arteriosus. According to TRANSFAC, *TMEM260* has been predicted to be regulated by GATA6 (7). *TMEM260* is predicted to be one of 1442 target genes for GATA6 predicted using TRANSFAC. GATA6 is a key regulator in heart development, which provides some clues in underlining the role of impaired *TMEM260* inducing congenital heart disease (7), but the binding site and motif of GATA6 on *TMEM260* are not yet clear. Further studies on the molecular function of *TMEM260* are therefore needed.

Membrane proteins are a significant portion of the proteome, accounting for ~30% of all proteins (12, 13). Most membrane proteins from mammalian cells are well characterized in terms of biological function and structural identification. They can be classified into various categories, including receptors such as G protein-coupled receptors, tyrosine kinase receptors, glucose transporters, ion channels, anchorage proteins (e.g., connexin, cadherin, and occludin), and structural proteins. These molecules play important roles in the formation of structures, such as caveolae, rafts, and filopodia, and in the regulation of endocytosis (14). Despite the well-characterized nature of some membrane proteins, many transmembrane proteins are poorly described and grouped under the TMEM family. To be considered a member of this family, proteins must contain at least one putative transmembrane segment that fully or partially spans biological membranes (14). While functions have been identified for some of these proteins, the majority remain poorly characterized, making it challenging to understand their mechanisms of action. Further research into this protein family could lead to the discovery of novel targets for developing new therapies. *TMEM260* is a member of the TMEM family, and little is currently known about its role. Its protein structure and function are poorly understood, and the identification of a pathogenic site, in this case, is of significant importance for exploring the structure and function of this protein.

The findings of this case report highlight the importance of considering genetic mutations as a potential cause of complex congenital heart disease. The novel homozygous mutation in the *TMEM260* gene identified in this patient presents a unique contribution to the field and expands the understanding of the genetic basis of congenital heart disease. The results also emphasize the need for comprehensive genetic analysis in the evaluation of congenital heart disease, particularly in cases where other potential causes have been ruled out. Moreover, the results of this case report emphasize the need for genetic counseling for families who are carriers of this mutation as it may affect future pregnancies and the development of offspring. Further research is needed to determine the frequency and clinical significance of this mutation in other populations and to fully understand the impact of this genetic alteration on the development of congenital heart disease.

## 4. Conclusion

Our case report highlights the importance of the *TMEM260* gene in the development of both the cardiovascular and neurological systems. We have identified a novel homozygous variant in the *TMEM260* gene in a proband with severe congenital heart defects and cerebral atrophy. This expands the known variant spectrum of the *TMEM260* gene and supports the role of *TMEM260* in the development of CHD. Our findings emphasize the importance of genetic evaluation for familial CHD patients, as well as for patients with a positive history of adverse pregnancy outcomes or multiple defects. Further research on the mechanisms of action of *TMEM260* is necessary to improve our understanding of the genetic basis of CHD and to facilitate the development of more efficient therapies. Overall, our study contributes to the growing body of evidence linking the *TMEM260* gene with CHD and neurodevelopmental abnormalities.

## Data availability statement

The original contributions presented in the study are included in the article/supplementary material, further inquiries can be directed to the corresponding authors.

## Ethics statement

The studies involving human participants were reviewed and approved by Ethics Committee of West China Second Hospital of Sichuan University (2014-034). Written informed consent to participate in this study was provided by the participants' legal guardian/next of kin. Written informed consent was obtained from the individual(s), and minor(s)' legal guardian/next of kin, for the publication of any potentially identifiable images or data included in this article.

## Author contributions

MP, GL, KZ, YH, TW, and YL were the patient's physicians. SJ and PY reviewed the literature and contributed to manuscript drafting. MP and SJ performed the mutation analysis. PY and YL conceptualized and designed the study, coordinated and supervised data collection, and critically reviewed the manuscript for important intellectual content. YL, PY, and TW were responsible for the revision of the manuscript for important intellectual content. All authors issued final approval for the version to be submitted.
